# A reproducible, diagnostic-guided hybrid Laplace workflow for generalized linear mixed models

**DOI:** 10.1016/j.mex.2026.104173

**Published:** 2026-09-22

**Authors:** Mohammad M. Hamasha

**Affiliations:** Department of Industrial Engineering, Faculty of Engineering, The Hashemite University, Zarqa, 13133, Jordan

**Keywords:** Laplace approximation, Generalized linear mixed models, Adaptive gauss–Hermite quadrature, Approximation diagnostics, Reproducible statistical workflow

## Abstract

Laplace approximation is widely used for generalized linear mixed models because it is fast and compatible with automatic-differentiation software, yet its accuracy can deteriorate when clusters are weakly informative, outcomes are sparse, or conditional random-effect distributions are skewed, heavy-tailed, or poorly conditioned. This article presents a reproducible diagnostic-guided hybrid Laplace workflow. The method begins with an ordinary Laplace fit, assigns blockwise approximation-risk scores, and selectively upgrades only flagged random-effect blocks using higher-fidelity corrections such as adaptive Gauss–Hermite quadrature, importance sampling, or skew-aware correction. This selectivity retains ordinary Laplace contributions for unflagged blocks but should not be interpreted as a demonstrated wall-clock speed-up: in the scalar benchmarks reported here, diagnostic overhead makes the hybrid slower than global 15-point adaptive Gauss–Hermite quadrature. A computational advantage is expected only in regimes where the cost of high-fidelity correction materially exceeds the cost of diagnostic screening and remains to be established there. In a rare-event Bernoulli random-intercept validation, the diagnostic score ranked absolute Laplace log-integral error well (Spearman ρ=0.84). Correcting the highest-risk third of blocks reduced the mean absolute error on flagged blocks from $5.27\;\times 10^{-2}$ under ordinary Laplace to $1.35\;\times 10^{-4}$ under 15-point adaptive Gauss–Hermite quadrature.

• Start from the standard Laplace fit already produced by routine GLMM software.
• Diagnose approximation risk block by block rather than replacing Laplace globally.
• Correct only flagged blocks and report an auditable trust summary.

## Graphical abstract

It summarizes the workflow as: baseline Laplace fit → blockwise diagnostics → thresholding → selective fallback correction → auditable reporting dashboard.

**Figure:**
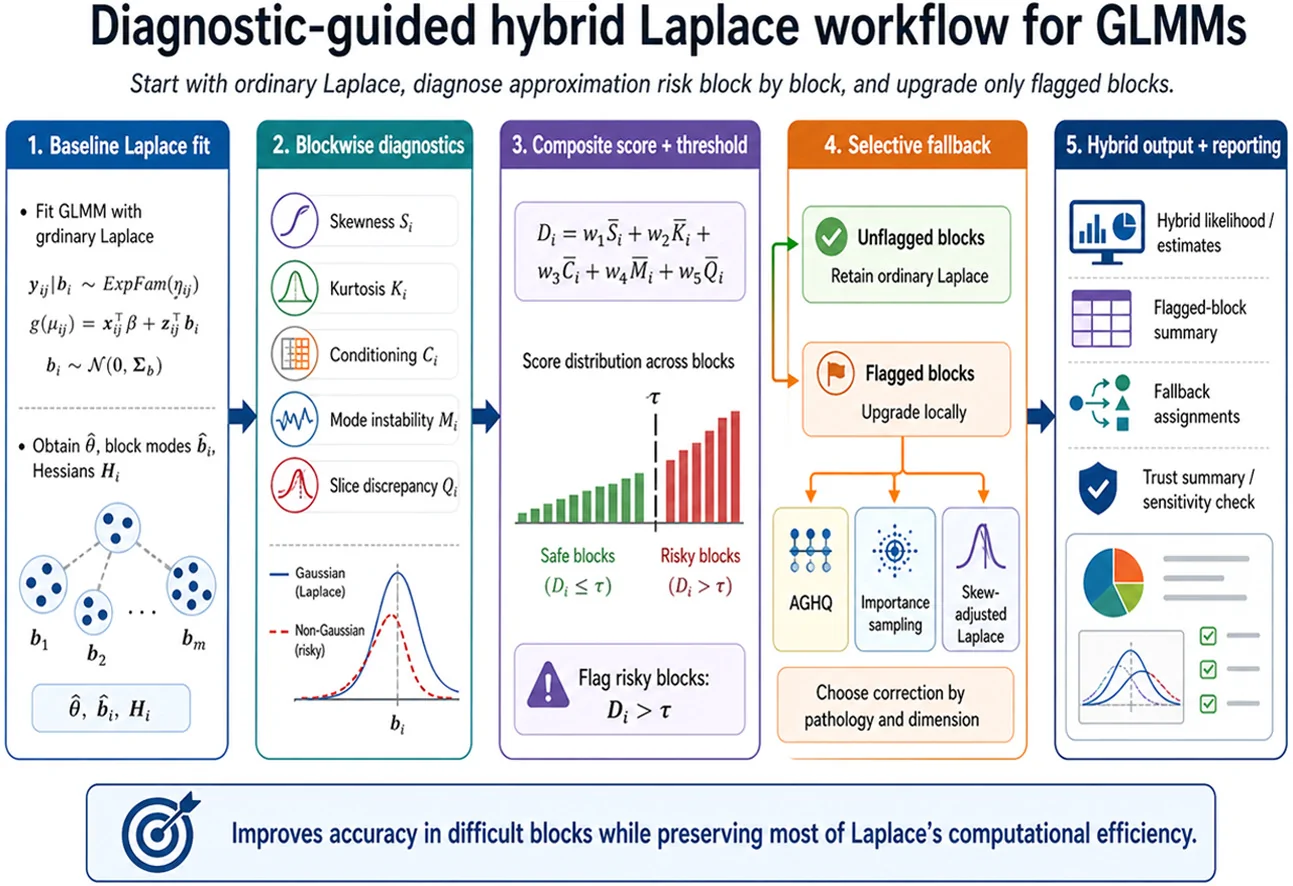

## Specifications table

**Table:** 

Subject area	Statistics / biostatistics / computational methods
More specific subject area	Approximate likelihood inference for generalized linear mixed models and latent Gaussian models
Name of your method	Diagnostic-guided hybrid Laplace fitting
Name and reference of original method	First-order Laplace approximation for latent-variable integration in mixed models, with adaptive Gauss–Hermite quadrature and related higher-fidelity corrections as candidate fallbacks [[1], [2], [3]]
Resource availability	No external dataset is required. The submitted supplementary reproducibility bundle contains the executable simulation, baseline fitting, diagnostic, correction, sensitivity, and timing code; a README; and machine-readable block-level and summary outputs. The validation data are regenerated deterministically from the model settings reported below. The separate graphical abstract file is included with the submission package.

## Value of the method

• The method retains ordinary Laplace contributions for diagnostically acceptable blocks and escalates only the unreliable blocks. This is selective computation, not a demonstrated wall-clock speed-up; diagnostic overhead can offset the savings from avoiding high-fidelity correction on every block.
• The workflow is implementation-oriented: it specifies required inputs, default diagnostic ingredients, an explicit directional recipe, a transparent thresholding rule, a pathology-to-fallback map, and a minimum reporting standard.
• The method produces not only corrected likelihood contributions but also auditable outputs—flagged-block counts, fallback assignments, optional trust summaries, and sensitivity summaries—that are useful for routine applied GLMM work.
• Relative to the status quo of using ordinary Laplace everywhere, the method adds an explicit decision layer that documents when the default approximation was retained and when it was escalated.

## Background

Laplace approximation is widely used because it turns otherwise intractable latent-variable integrals into local quadratic calculations around conditional modes [1,4]. In GLMMs, this makes routine likelihood-based fitting feasible at scales where global quadrature or full Bayesian sampling may be too expensive [5,6]. The drawback is equally well known: when cluster information is weak, outcomes are highly discrete, or the conditional random-effect distribution is skewed, heavy-tailed, or multimodal, naive Laplace can bias marginal likelihood contributions and understate uncertainty [[7], [8], [9], [10], [11], [12], [13]].

Existing alternatives improve accuracy but are often expensive when applied globally. Adaptive Gaussian quadrature is accurate in low dimension but does not scale well with random-effect dimension [3,14,15]. Importance sampling and Bayesian reference fits can be highly effective, but they are not always the preferred first-line tools for routine frequentist fitting [[16], [17], [18], [19]]. Recent work has also produced skew-aware corrections, probabilistic diagnostics, and computable error bounds for Laplace approximations [[20], [21], [22], [23], [24]]. The practical gap is not a lack of ideas; it is the absence of a simple workflow that tells users *when* the baseline Laplace fit should be trusted and *which* local correction should be used when it should not Tables 1 and 5.

**Table 1:** Minimum inputs, outputs, and suggested defaults for a first implementation.

Item	Recommended default	Purpose
Directional set size L	–5 directions per block	Captures leading non-Gaussian shape at modest cost
Diagnostic weights $w_{1},\ldots ,w_{5}$	Equal weights	Transparent screening rule before calibration
Threshold rule	Calibrated τ if available; otherwise top 10–30% of blocks or a fixed pilot threshold	Controls fraction of corrected blocks
Fallback for scalar / low-dimensional blocks	- or 15-point AGHQ	Accurate local correction when $q_{i}$ is small
Fallback for moderate-dimensional blocks	Importance sampling around the Laplace mode/covariance	Avoids global quadrature explosion
Refitting mode	One-step corrected update	Stable default for first implementation
Reporting bundle	Flagged-block list, trust scores, threshold used, sensitivity run	Makes approximation risk auditable

The aim of this article is therefore pragmatic. Rather than replacing Laplace everywhere, we describe a reproducible customization of standard Laplace-based GLMM fitting that adds blockwise diagnostics, selective fallback, and structured reporting to the existing computational pipeline. The contribution is not a new asymptotic theorem or a new quadrature family; it is an implementation-ready decision workflow that turns a familiar approximation into an auditable adaptive procedure. That framing is important because the scientific burden of proof is correspondingly practical: the method should be judged by whether its diagnostics discriminate harmful blocks, whether its local corrections materially reduce approximation error, whether its thresholding rule is calibratable, whether its outputs are reproducible, and whether, in the intended higher-cost correction regimes, its total computational overhead can fall below that of a global high-fidelity correction; this is an operating target rather than an outcome established by the present scalar experiments.

## Method details

### Pre-implementation requirements

#### Scope and intended use

This workflow is intended for GLMMs or closely related latent Gaussian models in which the fitted software can supply conditional random-effect modes, negative Hessians, and directional derivative evaluations. The method is particularly useful when ordinary Laplace is attractive computationally but may be unreliable because of sparse binary outcomes, low-information clusters, strong skewness, or optimizer instability. The workflow is designed as a *decision layer* over an existing Laplace-based fit rather than as a replacement fitting engine.

### Software assumptions

The implementation assumes access to the following quantities from a baseline Laplace fit:

1. parameter estimates ${\hat{\theta }}_{\text{LA}}$,
2. blockwise conditional modes ${\hat{b}}_{i}$,
3. negative Hessians $H_{i}=-\nabla^{2}g_{i}\left({\hat{b}}_{i};\theta \right)$,
4. the ability to evaluate the conditional log-integrand $g_{i}\left(b_{i};\theta \right)$ along chosen directions, and
5. optional multiple-start optimizer output for each block.

No new model-fitting engine is required; the workflow is designed as a wrapper around an existing Laplace-based fit.

### Inputs, outputs, and defaults

#### Minimal software contract

Table 2 states the smallest set of callable components needed for a reproducible first implementation. This explicit contract is helpful both for software developers and for MethodsX readers who want to reproduce the workflow in another stack.

**Table 2:** Minimal software contract for a first implementation.

Component	Required return values	Practical note
Baseline Laplace fit	${\hat{\theta }}_{\text{LA}}$, ${\hat{b}}_{i}$, $H_{i}$, optimizer status	Usually already available in TMB / glmmTMB-style software
Directional evaluator	$g_{i}\left({\hat{b}}_{i}+H_{i}^{-1/2}tv;\theta \right)$ for chosen (t,v)	Can be supplied by automatic differentiation or direct objective evaluation
Multiple-start wrapper	Alternative block modes and convergence status	Optional but useful when multimodality is suspected
Correction engine	AGHQ, importance sampling, or skew-aware correction for flagged blocks	A first release only needs one correction per pathology class
Report generator	Blockwise diagnostics, flagged set, fallback assignments, trust scores, timing summary	These artifacts should be saved, not only printed to screen

**1. Baseline fit and notation**

Consider clustered observations $y=(y_{1},\ldots ,y_{m})$ with random-effect blocks $b=(b_{1},\ldots ,b_{m})$, where $b_{i}\in R^{q_{i}}$. Let θ collect fixed effects and variance parameters. The blockwise conditional log-integrand is

(1) $$g_{i}\left(b_{i};\theta \right)=\text{log}p\left(y_{i}|b_{i},\theta \right)+\text{log}p\left(b_{i}|\theta \right),$$

and the marginal log-likelihood is

(2) $$l\left(\theta \right)=\sum_{i=1}^{m}\text{log}\int \text{exp}\left\{g_{i}\left(b_{i};\theta \right)\right\}\;db_{i}.$$

If ${\hat{b}}_{i}\left(\theta \right)$ is the conditional mode and $H_{i}\left(\theta \right)=-\nabla^{2}g_{i}\left({\hat{b}}_{i};\theta \right)$ is the negative Hessian, then the ordinary first-order Laplace contribution is

(3) $${\tilde{l}}_{i}^{\text{LA}}\left(\theta \right)=g_{i}\left({\hat{b}}_{i};\theta \right)+\frac{q_{i}}{2}\text{log}\left(2\pi \right)-\frac{1}{2}\text{log}\left|H_{i}\left(\theta \right)\right|.$$

The proposed workflow starts from a standard Laplace fit and reuses quantities already available in TMB/glmmTMB-style software: ${\hat{\theta }}_{\text{LA}}$, blockwise conditional modes ${\hat{b}}_{i}$, Hessians $H_{i}$, optimizer diagnostics, and automatic-differentiation access to directional derivatives [5,6].

**2. Blockwise diagnostics**

The diagnostic stage operates in standardized local coordinates

(4) $$z_{i}=H_{i}^{1/2}\left(b_{i}-{\hat{b}}_{i}\right).$$

Expanding the log-integrand around the conditional mode gives

(5) $$g_{i}\left(b_{i};\theta \right)=g_{i}\left({\hat{b}}_{i};\theta \right)-\frac{1}{2}z_{i}^{⊤}z_{i}+\frac{1}{6}T_{3,i}\left[z_{i},z_{i},z_{i}\right]+\frac{1}{24}T_{4,i}\left[z_{i},z_{i},z_{i},z_{i}\right]+\cdots ,$$

where $T_{3,i}$ and $T_{4,i}$ represent standardized third- and fourth-order departures from the local Gaussian approximation.

More precisely, if $\nabla^{r}g_{i}\left({\hat{b}}_{i}\right)$ is the symmetric rth derivative tensor and $A_{i}=H_{i}^{-1/2}$, then $T_{r,i}\left[z_{1},\ldots ,z_{r}\right]=\nabla^{r}g_{i}\left({\hat{b}}_{i}\right)\left[A_{i}z_{1},\ldots ,A_{i}z_{r}\right]$ for r=3,4; brackets denote multilinear tensor contraction. This is the standardized derivative convention used in higher-order Laplace expansions [4].

Five ingredients are combined:

(6) $$S_{i}=∥T_{3,i}∥\;\;\;\;\left(\text{standardized}\;\text{skewness}\;\text{indicator}\right),$$

(7) $$K_{i}=∥T_{4,i}∥\;\;\;\;\left(\text{standardized}\;\text{kurtosis}\;\text{indicator}\right),$$

(8) $$C_{i}=\text{log}\kappa \left(H_{i}\right)\;\;\;\;\left(\text{curvature}\;\text{conditioning}\right),$$

(9) $$M_{i}=\underset{r,s}{\text{max}}∥{\hat{b}}_{i}^{\left(r\right)}-{\hat{b}}_{i}^{\left(s\right)}∥\;\;\;\left(\text{optimizer}\;\text{instability}\;\text{under}\;\text{multiple}\;\text{starts}\right),$$

(10) $$Q_{i}=\text{slice}\;\text{discrepancy}\;\text{around}\;{\hat{b}}_{i}\;\;\;\left(\text{shape}\;\text{mismatch}\;\text{along}\;\text{low}-\text{dimensional}\;\text{slices}\right).$$

In high-dimensional blocks, full derivative tensors are unnecessary. A practical implementation selects a small set of orthonormal directions $V_{i}=\left\{v_{i1},\ldots ,v_{iL}\right\}$, for example the leading eigenvectors of $H_{i}^{-1}$ plus one or two random directions, and computes one-dimensional slices

(11) $$h_{il}\left(t\right)=g_{i}\left({\hat{b}}_{i}+H_{i}^{-1/2}tv_{il};\theta \right)-g_{i}\left({\hat{b}}_{i};\theta \right).$$

Here $H_{i}^{-1/2}$ denotes the symmetric positive-definite matrix square root obtained from $H_{i}=U_{i}\Lambda_{i}U_{i}^{⊤}$ as $U_{i}\Lambda_{i}^{-1/2}U_{i}^{⊤}$; it is not a Cholesky factor. The leading eigenvectors are those of $H_{i}^{-1}$ (equivalently, the smallest-curvature eigenvectors of $H_{i}$), expressed in the standardized coordinate system. Random orthonormal directions complement these deterministic weak-curvature directions: they can reveal asymmetric or non-quadratic behavior that is oblique to the eigenbasis, while keeping the directional budget small. The random-number seed is stored in the model-level output. Directional cubic and quartic coefficients extracted from $h_{il}$ provide low-cost proxies for $S_{i}$ and $K_{i}$, while the discrepancy between $h_{il}\left(t\right)$ and the Gaussian benchmark $-t^{2}/2$ supplies $Q_{i}$.

For a first implementation, we recommend making these proxies fully explicit. Let G={±0.5,±1,±1.5,±2} be a default standardized grid and define

(12) $$s_{i\ell }=\left|\frac{d^{3}}{dt^{3}}h_{i\ell }\left(0\right)\right|,$$

(13) $$k_{i\ell }=\left|\frac{d^{4}}{dt^{4}}h_{i\ell }\left(0\right)\right|,$$

(14) $$q_{i\ell }=\frac{1}{\left|G\right|}\sum_{t\in G}\left|h_{i\ell }\left(t\right)+\frac{t^{2}}{2}\right|.$$

The blockwise summaries are then taken as robust aggregates across directions,

(15) $$S_{i}=\text{me}d_{\ell =1,\ldots ,L}s_{i\ell },\;\;\;\;K_{i}=\text{me}d_{\ell =1,\ldots ,L}k_{i\ell },\;\;\;\;\;Q_{i}=\text{me}d_{\ell =1,\ldots ,L}q_{i\ell },$$

which reduces sensitivity to a single noisy slice. A useful default directional set is: the leading one or two eigenvectors of $H_{i}^{-1}$, the direction of the conditional mode when ${\hat{b}}_{i}\neq 0$, and one random orthonormal direction when $q_{i}\geq 3$.

**2a. Finite-difference fallback when higher-order derivatives are unavailable**

Some production environments do not expose directional third- and fourth-order derivatives directly. In that case, the same slice-based workflow can still be implemented using symmetric finite differences with a small step size η in standardized coordinates. Because t is standardized by $H_{i}^{-1/2}$, η=0.5 is a scale-free starting value rather than a step on the raw random-effect scale. Evaluate η∈{0.25,0.5,1.0} and retain the largest value for which the derivative proxies are stable to a prespecified relative tolerance (10% is a practical pilot default); reduce η if the corresponding raw displacement $∥H_{i}^{-1/2}\eta v∥$ leaves the locally supported region. For a slice h(t) centered at the conditional mode,

(16) $$h^{\left(3\right)}\left(0\right)\approx \frac{h\left(-2\eta \right)-2h\left(-\eta \right)+2h\left(\eta \right)-h\left(2\eta \right)}{2\eta^{3}},$$

(17) $$h^{\left(4\right)}\left(0\right)\approx \frac{h\left(-2\eta \right)-4h\left(-\eta \right)+6h\left(0\right)-4h\left(\eta \right)+h\left(2\eta \right)}{\eta^{4}}.$$

This fallback is not as stable as direct automatic differentiation, but it is often adequate for ranking blocks by approximation risk. For reproducibility, the report states whether the reported diagnostics were obtained by automatic differentiation, finite differences, or a mixture of both.

**3. Composite score and thresholding**

Because $S_{i}$, $K_{i}$, $C_{i}$, $M_{i}$, and $Q_{i}$ are on different scales, each quantity is normalized within the fitted model using the corresponding across-block median:

(18) $${\bar{S}}_{i}=\frac{S_{i}}{\text{med}\left(S_{\cdot }\right)+\varepsilon },\;{\bar{K}}_{i}=\frac{K_{i}}{\text{med}\left(K_{\cdot }\right)+\varepsilon },\;{\bar{C}}_{i}=\frac{C_{i}}{\text{med}\left(C_{\cdot }\right)+\varepsilon },$$

with analogous definitions for ${\overset{‾}{M}}_{i}$ and ${\overset{‾}{Q}}_{i}$, and a small ε>0 to avoid division by zero. We use $\varepsilon =10^{-10}$ as the reproducible default; if the objective is evaluated at markedly different numerical precision, ε may instead be set to ten times the solver’s absolute tolerance. The composite diagnostic score is

(19) $$D_{i}=w_{1}{\overset{‾}{S}}_{i}+w_{2}{\overset{‾}{K}}_{i}+w_{3}{\overset{‾}{C}}_{i}+w_{4}{\overset{‾}{M}}_{i}+w_{5}{\overset{‾}{Q}}_{i},$$

where the weights are nonnegative and sum to one. Equal weights are a reasonable default. When repeated use is expected within a model class, the weights and threshold can be calibrated against a reference approximation such as AGHQ or high-accuracy numerical integration.

The flagged set is

(20) $$S_{\tau }=\left\{i:D_{i}>\tau \right\}.$$

In routine use, τ can be chosen either from a calibration set (for example, targeting a fixed false-negative rate for harmful blocks) or from a heuristic rule such as flagging the top p% of blocks while the method is being piloted.

**3a. Calibration against a harmful-block criterion**

To move the method from a heuristic screening rule to a scientifically testable procedure, we recommend defining a blockwise reference error on a pilot set of blocks,

(21) $$\Delta_{i}^{\text{ref}}=\left|{\tilde{l}}_{i}^{\text{ref}}-{\tilde{l}}_{i}^{\text{LA}}\right|,$$

where ${\tilde{l}}_{i}^{\text{ref}}$ is a higher-fidelity reference contribution obtained by AGHQ, dense importance sampling, or high-accuracy numerical integration. A block is then called *harmful* if $\Delta_{i}^{\text{ref}}>\delta$ for a user-declared tolerance δ. The tolerance should be chosen on the scale relevant to the application: for example, in a pilot study one may set δ to a practically visible blockwise log-likelihood discrepancy or to the smallest error that induces non-negligible drift in fixed-effect estimates.

Given a pilot sample with labeled harmful and non-harmful blocks, threshold and weight calibration can be stated as an optimization problem rather than a subjective choice. One simple default is to choose (w,τ) to minimize

(22) $$L\left(w,\tau \right)=\lambda \;\text{FN}\left(w,\tau \right)+\left(1-\lambda \right)\;\frac{\left|S_{\tau }\right|}{m_{\text{pilot}}},$$

subject to $w_{j}\geq 0$ and $\sum_{j=1}^{5}w_{j}=1$, where FN(w,τ) is the false-negative rate for harmful blocks and λ∈[0,1] encodes the scientific preference for safety versus computational burden. In practice, a simple grid search over a small family of weight vectors is sufficient. When such calibration is not yet available, the chosen threshold is labeled as provisional, and a sensitivity analysis is reported around it.

**3b. Operational threshold protocol**

For applied work, the following threshold protocol is easy to reproduce: (i) draw a pilot subset of blocks spanning low, medium, and high values of $D_{i}$; (ii) compute a reference approximation for those blocks; (iii) define a harmful-block tolerance δ; (iv) choose (w,τ) by grid search under the loss above; (v) fix (w,τ) before running the full hybrid fit; and (vi) report one nearby threshold and one nearby weight vector as a sensitivity check. Use at least min(30,m) pilot blocks when feasible, with no fewer than five blocks from each tertile of the preliminary $D_{i}$ distribution; for m<30, use all blocks or repeated stratified resampling. Increase the pilot until the selected threshold and estimated harmful-block recall change by less than 0.05 under one additional stratified batch. This protocol turns threshold selection from an undocumented tuning choice into a transparent calibration step.

**3c. Relation to alternative approximation strategies**

The selective workflow occupies a middle ground between ordinary Laplace, global AGHQ, importance sampling, INLA-style approximation, and full sampling. Ordinary Laplace is fastest and easiest to deploy but offers no block-specific safeguard in difficult discrete-data regimes [7]. Global AGHQ can deliver excellent accuracy for scalar or very low-dimensional blocks, but its tensor-product cost grows rapidly with dimension [3,15]. Importance sampling is dimension-tolerant and provides a direct integration check, but proposal selection and Monte Carlo variability require monitoring. INLA and enhanced or skew-adjusted Laplace methods can improve accuracy systematically, although they entail a different approximation stack or correction model [16,17,22]. The proposed advantage is therefore operational rather than universal: it preserves an installed Laplace pipeline and allocates higher-fidelity computation only where diagnostics indicate risk. This selectivity is a workload-allocation property, not evidence of lower wall-clock time; in the present scalar benchmarks, diagnostic overhead makes the hybrid slower than global AGHQ. Its disadvantages are the calibration overhead, possible false negatives, and dependence on having an appropriate fallback for each flagged block.

**4. Selective fallback map**

For blocks not in $S_{\tau }$, the Laplace contribution in Eq. 3 is retained. Flagged blocks are reassigned to a more accurate local approximation according to the dominant pathology. Table 3 shows a practical default map.

**Table 3:** Default selective fallback policy.

Primary pathology	Typical indicator pattern	Default correction
Low-dimensional non-Gaussian shape	high ${\overset{‾}{S}}_{i}$ or ${\overset{‾}{K}}_{i}$, small $q_{i}$	AGHQ
Stable but skewed unimodal block	high ${\overset{‾}{S}}_{i}$, modest ${\overset{‾}{M}}_{i}$	skew-adjusted or enhanced Laplace
Moderate-dimensional unimodal block	elevated ${\overset{‾}{S}}_{i}$ or ${\overset{‾}{Q}}_{i}$, moderate $q_{i}$	importance sampling centered at the Laplace mode/covariance
Suspected multimodality or optimizer instability	large ${\overset{‾}{M}}_{i}$ and/or large ${\overset{‾}{Q}}_{i}$	multiple-start integration or local mixture correction
Ill-conditioned weak-information block	large ${\overset{‾}{C}}_{i}$ with flat slices	importance sampling or higher-fidelity local quadrature

The resulting hybrid objective is

(23) $$\tilde{l}\left(\theta \right)=\sum_{i\notin S_{\tau }}{\tilde{l}}_{i}^{\text{LA}}\left(\theta \right)+\sum_{i\in S_{\tau }}{\tilde{l}}_{i}^{\text{corr}}\left(\theta \right),$$

where ${\tilde{l}}_{i}^{\text{corr}}$ is the selected corrected block contribution.

For operational use, the fallback map should be dimension-aware as well as pathology-aware. A practical default policy is: use 11- or 15-point AGHQ when $q_{i}\leq 2$ and the dominant signal is non-Gaussian shape; use Laplace-centered importance sampling when $3\leq q_{i}\leq 6$ and the block appears unimodal but non-quadratic; and reserve multiple-start proposals or local mixture corrections for blocks with large $M_{i}$ or severe slice asymmetry that suggests unresolved multimodality. This explicit rule narrows the set of discretionary choices and makes cross-study comparison easier.

**5. Reporting summary and optional trust score**

At minimum, the workflow should return a blockwise reporting summary based on the diagnostic score $D_{i}$, the flagging decision, and the assigned fallback. For implementations that benefit from a single compact quality-control indicator, an *optional* blockwise trust score can be defined as

(24) $$T_{i}=\text{exp}\left(-\alpha D_{i}\right),\;\;\;\;\;0<T_{i}\leq 1,$$

with optional model-level aggregation

(25) $$T_{\text{model}}=\text{exp}\left[-\alpha \frac{1}{m}\sum_{i=1}^{m}D_{i}-\beta \frac{\left|S_{\tau }\right|}{m}\right].$$

Here α and β are user-calibrated scaling parameters. A convenient default is α=log(2)/τ, so that a block exactly at the flagging threshold receives score 0.5, and β=ατ=log(2) so that the model-level score is visibly penalized once a nontrivial fraction of blocks are flagged. These quantities are not posterior probabilities that Laplace is “correct”; they are implementation-facing summaries of approximation risk and may be omitted if a project prefers to report only the raw diagnostics and fallback map.

For reproducible reporting, we recommend always returning:

1. baseline Laplace estimates and hybrid estimates,
2. the number and fraction of flagged blocks,
3. the fallback type assigned to each flagged block or block stratum,
4. the distribution of $D_{i}$ together with either the optional trust score or a simpler diagnostic dashboard, and
5. one sensitivity check over an alternative threshold or weight setting.

**5a. Suggested machine-readable outputs**

To make the method easy to audit, we recommend writing three files for each fit: (i) a block-level table containing $D_{i}$, its diagnostic components, the fallback assignment, and, if used, the final trust score; (ii) a model-level summary with the threshold, weight vector, flagged fraction, timing information, and software versions; and (iii) a short human-readable report that includes one threshold sensitivity run. These artifacts make the approximation decision process inspectable after the fact.

**6. Practical implementation steps**

The method can be implemented as the following six-step workflow.

Step 1.Fit the GLMM using the ordinary Laplace approximation and store ${\hat{\theta }}_{\text{LA}}$, ${\hat{b}}_{i}$, $H_{i}$, and optimizer information.

Step 2.For each block, build a small directional set $V_{i}$ and compute $S_{i}$, $K_{i}$, $C_{i}$, $M_{i}$, and $Q_{i}$.

Step 3.Normalize the diagnostic ingredients, construct $D_{i}$ using Eq. 19, and determine $S_{\tau }$.

Step 4.Assign each flagged block to AGHQ, importance sampling, or a skew-aware correction using Table 3.

Step 5.Recompute the objective in Eq. 23; in software, a one-step corrected update is a stable default, while iterative re-diagnosis can be reserved for sensitivity analysis.

Step 6.Return parameter estimates, uncertainty summaries, the fallback map, optional trust scores, and machine-readable output files.

**7. Computational cost and expected operating regime**

The scientific case for the workflow depends first on diagnostic usefulness and accuracy gains; computational savings are an intended operating benefit that must be assessed empirically in the correction regime of interest. The present scalar benchmarks do not establish a speed advantage over global high-fidelity correction. The diagnostic pass requires only a small number of directional evaluations per block, so its extra cost is approximately

(26) $$C_{\text{diag}}\approx \sum_{i=1}^{m}L\;c_{i}^{\left(g\right)},$$

where $c_{i}^{\left(g\right)}$ is the cost of evaluating one directional slice of $g_{i}$. The correction stage scales with the flagged set only,

(27) $$C_{\text{corr}}\approx \sum_{i\in S_{\tau }}c_{i}^{\left(\text{corr}\right)},$$

so the workflow limits high-fidelity correction to the flagged set. Whether this selectivity reduces total wall-clock time depends on whether the correction costs avoided on unflagged blocks exceed the diagnostic, bookkeeping, and refit overhead introduced by the hybrid procedure. In first empirical studies, the paper should therefore report not only approximation improvements but also the fraction of corrected blocks and the wall-clock ratio

(28) $$R_{\text{time}}=\frac{\text{runtime}\;\text{of}\;\text{hybrid}\;\text{fit}}{\text{runtime}\;\text{of}\;\text{baseline}\;\text{Laplace}\;\text{fit}},$$

with comparison to at least one global high-fidelity strategy where feasible. A useful operating target—rather than a result established here—is that the hybrid method recover most of the reference accuracy while correcting far fewer than all blocks and, in regimes where per-block correction is sufficiently expensive, reduce total wall-clock time relative to a global high-fidelity strategy.

**8. Minimal pseudocode**

Input: fitted Laplace model, block modes ${\hat{b}}_{i}$, Hessians $H_{i}$, directional evaluator for $g_{i}$, threshold τ

For each block i: construct $V_{i}$ and compute $S_{i},K_{i},C_{i},M_{i},Q_{i}$

Normalize dia**g**nostics across blocks and compute $D_{i}$

If pilot references are available: calibrate (w,τ) against the harmful-block criterion.

Flag blocks with $D_{i}>\tau$

For each flagged block: choose fallback method from Table 3.

Recompute the hybrid objective using corrected contributions only for flagged blocks.

Write a block-level report, a model-level summary, and a short sensitivity report.

Return estimates, optional trust scores, flagged-block summary, runtime ratio, and reproducibility artifacts.

**9. Reproducibility bundle**

The workflow is easiest for other groups to reuse when paired with a minimal reproducibility package. Table 4 gives a compact checklist for reuse without requiring a large software release.

**Table 4:** Minimal reproducibility bundle recommended for reuse.

Artifact	Minimum content
Simulation script	Generates the validation example with fixed seed and user-visible parameter settings
Baseline fit script	Runs the ordinary Laplace fit and saves ${\hat{\theta }}_{\text{LA}}$, ${\hat{b}}_{i}$, and $H_{i}$
Diagnostic script	Computes $S_{i}$, $K_{i}$, $C_{i}$, $M_{i}$, $Q_{i}$, $D_{i}$, and the flagged set
Correction script	Applies AGHQ or the chosen fallback to flagged blocks only
Output files	Block-level report, model-level summary, timing summary, and one sensitivity run
README	Software versions, seed, threshold, weight vector, and exact command sequence

**Table 5:** Practical troubleshooting notes for first implementations.

Issue	Likely cause	Practical response
Almost all blocks are flagged	Threshold too low or diagnostics not normalized robustly	Re-check within-model normalization; pilot a higher threshold or percentile rule
No blocks are flagged in known hard regimes	Threshold too high or directional set too small	Increase L, inspect slice plots, or calibrate τ against a low-dimensional reference fit
Unstable fallback estimates	Importance proposal too narrow or multimodality unresolved	Inflate proposal covariance, add multiple starts, or switch to low-dimensional quadrature where feasible
Large trust-score swings across nearby fits	Threshold jitter or weight sensitivity	Report a sensitivity run with one alternative threshold and one alternative weight vector
High computational burden after fallback	Too many blocks corrected or fallback too expensive for $q_{i}$	Use one-step correction, group similar blocks, or reserve AGHQ for scalar / very low-dimensional blocks

In a small first release, it is sufficient for these scripts to run the illustrative validation example from start to finish and reproduce the values reported in this article. That level of transparency is often more useful to readers than a large but under-documented repository.

### Implementation notes and troubleshooting

▓

## Method validation

### Proof-of-concept scalar validation example

MethodsX asks method papers to show evidence of efficiency and/or comparison with pre-existing practice, so the validation is framed as a direct comparison between the status quo (ordinary Laplace for every block) and the proposed selective-upgrade workflow. This article provides a deliberately compact *proof-of-concept* validation in a scalar Bernoulli random-intercept setting; it is intended to verify that the decision workflow behaves sensibly in a transparent hard regime, not to claim exhaustive validation across all GLMM classes. We considered a scalar Bernoulli random-intercept block in a deliberately difficult rare-event regime. Conditional on a cluster-level random intercept b, observations satisfy

(29) $$Y_{ij}|b∼\text{Bernoulli}\left(\text{logi}t^{-1}\left(\beta_{0}+b\right)\right),$$

with $\beta_{0}=-4$ and $b∼N(0,\sigma^{2})$, σ=3. We evaluated cluster sizes n∈{4,6,8} and all success counts y∈{0,…,n}. For each scalar block, the reference marginal log-integral was obtained by high-accuracy numerical integration, the baseline approximation was ordinary Laplace, and the fallback correction was 15-point adaptive Gauss-Hermite quadrature. A simplified one-dimensional version of the proposed diagnostic was used: $D_{i}$ was formed from within-grid normalized skewness, kurtosis, and slice discrepancy with equal weights.

Across the 21 validation blocks, the composite score ranked blocks by absolute Laplace log-integral error with Spearman ρ=0.84. Using an example threshold τ=1.5, the method flagged 7 of 21 blocks (33.3%). On those flagged blocks, mean absolute log-integral error dropped from $5.27\;\times 10^{-2}$ under ordinary Laplace to $1.35\;\times 10^{-4}$ under 15-point AGHQ. Table 6 now reports all 21 blocks rather than selected representatives. Threshold sensitivity was modest: τ=1.25, 1.50, and 2.00 flagged 8, 7, and 4 blocks, respectively; their mean flagged-block Laplace errors were $5.33\;\times 10^{-2}$, $5.27\;\times 10^{-2}$, and $5.26\;\times 10^{-2}$, and their corrected errors were $1.19\;\times 10^{-4}$, $1.35\;\times 10^{-4}$, and $2.22\;\times 10^{-4}$. This compact example therefore establishes two practically important points: the diagnostic score discriminates between easier and harder scalar blocks, and selective local correction can sharply reduce error on flagged blocks while retaining ordinary Laplace contributions for two thirds of blocks. This demonstrates selective allocation of correction effort, not a wall-clock speed-up in the present scalar benchmark.

**Table 6:** Complete results for all 21 Bernoulli validation blocks. Here D is the simplified scalar composite score, and “Flagged” uses the example threshold τ=1.5.

Cluster size n	Successes y	D	|Laplace error|	|15-point AGHQ error|	Flagged
4	0	3.790	0.05888	0.000326	Yes
4	1	1.100	0.05812	0.000008	No
4	2	0.713	0.04989	0.000001	No
4	3	0.737	0.05056	0.000001	No
4	4	1.363	0.05799	0.000007	No
6	0	4.299	0.05289	0.000288	Yes
6	1	1.549	0.05340	0.000016	Yes
6	2	0.699	0.04206	0.000003	No
6	3	0.513	0.03604	0.000000	No
6	4	0.529	0.03637	0.000000	No
6	5	0.713	0.04319	0.000004	No
6	6	1.785	0.05372	0.000016	Yes
8	0	4.590	0.04704	0.000249	Yes
8	1	1.785	0.05116	0.000024	Yes
8	2	0.670	0.03894	0.000005	No
8	3	0.552	0.03119	0.000001	No
8	4	0.398	0.02805	0.000000	No
8	5	0.408	0.02822	0.000000	No
8	6	0.568	0.03179	0.000001	No
8	7	0.743	0.04018	0.000006	No
8	8	2.005	0.05170	0.000025	Yes

### Additional Poisson random-intercept validation

To test a second response family, we also evaluated 21 Poisson random-intercept blocks with $Y|b∼\text{Poisson}\{n\text{exp}(\beta_{0}+b)\}$, $\beta_{0}=-2.5$, σ=1.8, exposures n∈{2,4,6}, and counts y=0,…,6. High-accuracy one-dimensional integration again supplied the reference. The diagnostic–error rank correlation was ρ=0.35, weaker than in the Bernoulli regime and therefore informative about model-specific calibration. At τ=1.5, 5 of 21 blocks (23.8%) were flagged; mean absolute error on those blocks decreased from $1.60\;\times 10^{-2}$ under Laplace to $1.21\;\times 10^{-5}$ under 15-point AGHQ. At τ=1.25, 7 blocks were flagged and corrected mean error was $1.68\;\times 10^{-5}$; τ=2.0 flagged none. This result supports the correction step but also shows why a threshold calibrated in one family should not be transferred mechanically to another. Complete Poisson block results are supplied in outputs/validation_blocks.csv.

### Wall-clock comparison

Wall-clock timings were obtained by repeating each complete 21-block calculation 200 times in Python 3.12.13/SciPy 1.17.0 on the submission build environment. For Bernoulli blocks, baseline Laplace, the diagnostic-guided hybrid, and global 15-point AGHQ required 0.0040, 0.0086, and 0.0069 s per full calculation, respectively; for Poisson blocks the corresponding times were 0.0043, 0.0081, and 0.0068 s. Thus this transparent scalar reference implementation is approximately 1.90–2.14 times slower than baseline Laplace and, because diagnostic overhead dominates these tiny one-dimensional problems, is not faster than global AGHQ. This is an important boundary condition rather than a claimed speed advantage. More generally, a wall-clock advantage over global correction can arise only when the cumulative cost of high-fidelity corrections avoided on unflagged blocks exceeds the diagnostic-screening, bookkeeping, and refit overhead introduced by the hybrid workflow. That condition is not met in these tiny one-dimensional examples, where 15-point AGHQ is itself inexpensive. It is expected to become more plausible as per-block correction cost rises with dimension or correction complexity, but this expectation remains to be demonstrated. The supplied timing script permits direct benchmarking in that intended regime.

### What is empirically established here

The validation supports a bounded but useful claim in the demonstrated scalar Bernoulli and Poisson random-intercept settings. Three findings should be separated: (i) the diagnostic workflow can identify higher-risk blocks, with substantially stronger diagnostic–error ranking in Bernoulli than in Poisson; (ii) selective 15-point AGHQ correction can materially reduce approximation error on flagged blocks; and (iii) the present wall-clock benchmarks do not demonstrate a computational speed advantage over global high-fidelity correction. The method therefore demonstrates diagnostic discrimination and selective error reduction, while computational speed-up remains an expectation for higher-cost correction regimes. These scalar experiments do *not* by themselves establish performance for higher-dimensional random-effect structures, overdispersed counts, or severe multimodality; those settings require the extension benchmarks outlined below.

### Recommended extension scenarios for broader deployment

For deployment in a broader GLMM pipeline, the next benchmark scenarios should be Bernoulli random intercept plus slope models, overdispersed count models, and moderate-dimensional correlated blocks in which full AGHQ is no longer attractive. In those settings, the same reporting bundle proposed here should be retained: screening performance of $D_{i}$, movement of parameter estimates toward a reference fit, fraction of corrected blocks, and runtime ratio relative to ordinary Laplace, ideally with comparison against at least one global higher-fidelity strategy. These future benchmarks should test explicitly whether the high-fidelity correction costs avoided on unflagged blocks exceed the diagnostic and hybrid overhead. The present Poisson experiment broadens response-family coverage, whereas random-slope and correlated multivariate blocks remain the priority for subsequent validation.

## Limitations

The proposed workflow has several limitations. First, derivative-based local diagnostics cannot by themselves resolve severe multimodality; in those cases, multiple-start searches or mixture-based corrections remain necessary. Second, threshold calibration is application-dependent: aggressive thresholds increase computation, while conservative thresholds may miss problematic blocks. Third, no single fallback correction dominates across all dimensions and model classes. Fourth, any optional trust score is a calibrated reporting summary, not a formal guarantee. Fifth, the current validation is limited to scalar Bernoulli and Poisson random-intercept examples, so applications with higher-dimensional random effects, overdispersed counts, or severe multimodality should be accompanied by a small problem-specific calibration study before routine use. Sixth, the current scalar wall-clock benchmarks do not show a speed advantage over global AGHQ; any computational benefit should therefore be treated as an intended operating property to be tested in higher-cost correction regimes. Finally, the method is best viewed as a decision layer on top of existing approximation tools; if the underlying candidate corrections are poorly tuned, the hybrid workflow will inherit that weakness.

## Ethics statements

This article describes a computational method and uses only simulated data in the validation example. No human participants, animal experiments, or social-media data were involved.

## Supplementary material and/or additional information optional

The separate graphical abstract file Graphical_Abstract_Laplace.png is included. The supplementary reproducibility bundle also includes validation_workflow.py, run_validation.sh, README.txt, outputs/validation_blocks.csv, and outputs/validation_summary.json. Together they regenerate both validation families, all diagnostic scores, 15-point AGHQ corrections, threshold-sensitivity summaries, and wall-clock comparisons.

## Declaration of competing interest

The author declares that he has no known competing financial interests or personal relationships that could have appeared to influence the work reported in this paper.

## Data Availability

Data will be made available on request.
